# Devolution and grant-in-aid design for the provision of impure public goods

**DOI:** 10.1186/s40064-016-1919-9

**Published:** 2016-03-05

**Authors:** Laura Levaggi, Rosella Levaggi

**Affiliations:** Faculty of Science and Technology, Free University of Bolzano-Bozen, Piazza Università 1, 39100 Bolzano-Bozen, Italy; Department of Economics and Management, University of Brescia, Via S. Faustino, 74b, 25122 Brescia, Italy

**Keywords:** Devolution, Impure public goods, Spillovers, Grants-in-aid, H71, H72

## Abstract

Traditional fiscal federalism theory postulates that devolution for the provision of local public goods increases welfare. However, most of the services offered at local level are local impure public goods whose characteristics may prevent devolution from being efficient. Our paper shows that devolution is the optimal choice only for local impure public goods. For an environment characterised by coordination and asymmetry of information problems, we propose the optimal grants-in-aid formula that Central Government should use to reduce welfare losses and we compare it with what suggested by the mainstream literature. Finally, we show under which conditions devolution should be preferred to a centralised solution. From a policy point of view, our paper may explain the heterogeneity in the choices made by countries in terms of devolution in the provision of merit and impure public goods.

## Background

The process of decentralisation in decision making has received increasing attention in the past few years. In transition economies the break-up of centralised decision-making has required new systems of governance; in Eastern European and Latin American countries (Weisner 2003) more autonomy is demanded by lower tiers, even though its effects on economic growth and regional income disparities remain a controversial issue (Barrios and Strobl 2009; Sacchi and Salotti 2014; Sorens 2014); at EU level the discussion on the functions that should be centralised is still open (Thieben 2003; Tanzi 2009; Vaubel 2009).

The nature of services produced and financed by the public sector is changing: nowadays most services provided are local impure public goods with spillovers.^1^ Examples are health care and education, local transport, sporting facilities, waste disposal. These services generate utility directly to users (as private goods do), but also indirectly to non users (as local public goods do). Their beneficial effect is usually not confined to users in a specific jurisdiction and this why spillovers arise. According to OECD statistics, in 2014 public expenditure for health, and education accounted for about 12.2 % of total GDP, in the US the share is even higher (OECD 2016).

For these services, there is no consensus in the literature on which is the most appropriate government tier to deliver the good.^2^ The institutional setting is quite heterogeneous among countries. Dziobek et al. (2011) examine the fiscal decentralisation index for several countries and observe a large variation that does not simply depends on economic or geographical factors; for education Turati et al. (2011) show a quite heterogeneous picture, which is even greater for health and social care (Costa-Font and Greer 2013). In Alcidi et al. (2014) several measures of fiscal decentralisation are studied for the European Union, showing that different decentralisation strategies have been adopted, even within the same country. For Europe as a whole fiscal decentralisation (defined as the ratio of local expenditure to total expenditure) is around 20 % for health 40 % for education and 80 % for environment protection. Some countries are below all these levels (Austria, Belgium, Germany, Ireland, Luxembourg, Macedonia, Slovenia), a second group are above the average (Lithuania, Poland) while a third group of countries are well above the limit for some services and below for others.^3^

The reason for this observed heterogeneity is that the determination of the optimal quantity to be produced is more complex than for public goods. For the latter, the quantity supplied and financed also represents the quantity consumed by all the individuals in the community. For impure public goods a pseudo demand exists, and Government should use indirect instruments (such as subsidises to the price) to match demand with supply and to produce the optimal quantity. However, the information necessary to achieve a First Best (FB) result is usually not accessible and only second best solutions can be attained.

The traditional literature on fiscal federalism (Oates 1972; Tresch 2002) argue that the allocation of functions between Central and local Governments should follow efficiency principles. Production should be assigned to the tier which is better informed on local preferences, while Central Government (CG) may use grants for equity and efficiency reasons. Second generation models^4^ suggest that the success of fiscal federalism depends on the information the agents possess about specific parameters (Akai and Mikami 2006; Levaggi 2002; Wildasin 2004; Snoddon and Wen 2003) and on the level of coordination in the actions of the different agents (Besley and Coate 2003; Köthenbürger 2008; Petretto 2000; Ogawa and Wildasin 2009). Both issues have been widely studied by the literature, which shows the existence of a trade-off between autonomy and control.

In this article we compare centralisation (defined as the provision of goods at local level by CG) with devolution (defined as the provision of goods at local level by a local government) for the provision of an impure local public good with spillovers in an environment characterised by asymmetry of information. We show that the traditional “devolution is always welfare improving” is valid only in the absence of spillovers. When they do exist, the gain in utility derived from devolution must be sufficiently high to compensate the welfare loss deriving from asymmetry of information and lack of coordination. In this setting, we derive the optimal grants-in-aid formula and compare it with what suggested by the literature for local public goods.

From a policy point of view, our paper may explain why some countries have preferred centralisation to devolution for the provision of services such as education and health care. Calsamiglia et al. (2006) argue that it may depend on altruism; in this article we show that efficiency may be the driving factor. For services whose comparative advantage in being locally produced is limited, while their consumption produces spillovers, centralisation may be the second best choice.

The organisation of the paper is as follows: in “The model” the main features of the model and the FB are presented; in “Centralisation” the centralised solution is analysed, while in “Devolution” devolution is presented; they are then compared in “Centralisation versus devolution”; finally in “Conclusions” the conclusions are drawn.

## The model

A country, whose population is normalised to one, is divided into two local authorities $j\in \{ 1,2\}$, equal in everything but their preferences for the impure public good *y*.^5^ Each individual has an exogenous money income *M* in the range $[\underline{M},\overline{M}]$, whose distribution in each region has density function $\frac{1}{2} f(M)$. Then, total income is:

$$Y=\int _{\underline{M}}^{\overline{M}}M f(M)\text {{d}}M.$$

and total income in local authority *j* equals $Y_j=\frac{1}{2} Y$. Income is used to buy private commodities and one or zero units of an impure local public good *y*, whose user charge is equal to $p_{u}^{j}.$*y* is an impure public good, which means that it has a double effect on the utility function of each individual:

- as a private commodity;
- for the whole quantity that is produced.

Let us first consider the private characteristic. A good of quality $\theta _{l}$ produces an utility equal to $\alpha \theta$, where $\alpha$ is a taste parameter in the range $(0,\beta )$, equally distributed among the population. Therefore, if the difference $\alpha \theta _{l}-p_{u}^{j}$ is positive the consumer buys *y*, otherwise he does not buy the good and private utility is zero. To simplify the algebra, we assume that $\underline{M}>p^j_u$ for all *j*, i.e. everybody can afford *y*.

The nature of impure public good means that the commodity *y* accrues utility to users and non users; in our model this characteristics is represented by the term $z_{j}(\theta _{l}(Q_{j}+k\,Q_{-j}))$, where

1 $$Q_{j}=\int _{\frac{p_{u}^{j}}{\theta }}^{\beta }\frac{1}{2\beta }\,\text {d}\alpha =\frac{1}{2}\left( 1-\frac{p_{u}^{j}}{\theta \beta }\right) .$$

is the total quantity demanded in each community and $Q_{-j}$ is the quantity produced outside jurisdiction *j*. Let us examine each element: $z_{j}$ represents the relative importance that each community attaches to the public goods characteristic of *y*; the second important element is the parameter $k\in [0,1]$, which captures spillovers and represents the utility that consumers in jurisdiction *j* attribute to the production of good *y* outside their region. For $k=1,y$ is a national impure public good: *y* produces the same level of utility irrespective of its geographical location. For $k=0$ consumers only care about the quantity produced locally (impure local public good) and for $0<k<1$ we have a local impure public good with spillovers, i.e. consumers derive a higher level of utility from the quantity produced in their jurisdiction, but also care about the level produced elsewhere (see also Wildasin 2001, 2004). The preferences for the impure public good are linear and homogeneous within each local authority, but specific to each of them.^6^*y* can be supplied at central level by CG or by an autonomous lower government tier (LG) in each jurisdiction. In line with the fiscal federalism theory, *y* produces a level of utility $\theta _{C}=1$ if it is supplied by CG and $\theta _{L}=\theta >1$ when it is produced by LG. This classical hypothesis in the theory on fiscal federalism (Oates 1972; Tresch 2002) reflects the assumption that at local level the preferences for the community can be better matched by local supply.^7^ Finally, we assume that, while the cost to produce the good is *p*, users pay only a fraction $p^j_{u}$ of such cost. The difference is financed using income taxes at rate $t_{j}$ in each jurisdiction.

As in Besley and Coate (2003), we assume that utility is additive in its components and that taxation is linear. The use of a linear utility function allows concentrate the analysis on efficiency and to rule out distributional issues (Levaggi and Menoncin 2014).

The utility function for a representative individual living in community *j* is therefore written as:

2 $$\begin{aligned}&U^{j}\left( M;\alpha ;p_u^j;z_j\right) =M\left( 1-t_{j}\right) +\max \left( \alpha \theta _{l}-p^j_{u};0\right) +z_{j}\left( \theta _{l}\left( Q_{j}+k\,Q_{-j}\right) \right) ,\nonumber \\&\quad \qquad l =C\,\text{ or }\,L,\quad j=1,2. \end{aligned}$$

In this paper we take into consideration the problem of CG, that has to decide whether to delegate the supply of *y* to LGs and if so how to implement devolution. The objective of CG is to maximise the welfare of the population, defined as the aggregation of individual preferences. Asymmetry of information prevents CG to observe $z_1$ and $z_2$; CG therefore uses some expected value *z* for both, so that in general the goal is to maximise the following function:

3 $$\int _{\underline{M}}^{\overline{M}}\left( \int _{0}^{\beta } \left( U^{1}\left( M;\alpha ;p_u^1,z\right) +U^{2}\left( M;\alpha ;p_u^2;z\right) \right) \frac{1}{\beta } \text {d}\alpha \right) f(M)\text {d}M$$

by reducing the user charges $p^j_{u}$, $j=1,2$.

The traditional theory on fiscal federalism Oates (1972) postulates that when preferences are not homogeneous and the goods produced at local level have a higher level of utility, CG should always devolve any decision to lower tiers. However, coordination problems and information asymmetry may prevent the attainment of this objective when the good to be produced is, as in the present case, an impure public good with spillovers.

### First Best (FB)

To start with, let us define the solution for an ideal world with perfect information, where a benevolent social planner can supply the good of the highest quality, is able to observe the local preference parameters $z_{1}$, $z_2$ and can therefore supply the optimal amount of the local public good in each region.

This represents the ideal, FB allocation that will be used as a benchmark to evaluate the relative benefits of implementing either a centralised solution or devolution.

Let us examine (2) to understand the problem faced by the regulator. The quantity demanded depends on the private utility that each consumer receives from the good, but the benefit produced by that commodity also depends on the utility that the community as a whole derives from its provision.^8^ This causes the usual market failure that the literature on impure public goods has long studied and calls for an intervention of the public decision maker (Musgrave and Musgrave 1989). By subsidising *y*, the regulator reduces the user charge $p_u^j$, and increases demand, so that the optimal equilibrium between the benefit (public and private) produced by the good is equal to its marginal cost. If the regulator finances a fraction $(1-\rho _{j})$ of *p* in each region through a (national) linear income tax at rate *t*, the user charge becomes $p_u^j=\rho _j\,p$ and from (1) the budget constraint is equal to:

4 $$tY =\sum _{j=1}^{2}(1-\rho _{j})p Q_j = \frac{1}{2} \sum _{j=1}^{2}(1-\rho _{j})p \left( 1-\frac{\rho _j\,p}{\theta \beta }\right) .$$

The regulator has to find the optimal values of $\rho _1$ and $\rho _2$ for which total welfare in (3) is maximised. The optimal solution in terms of price subsidy $p(1-\rho _{j}^{FB})$, quantities produced in each region $Q_j^{FB}$, total quantity $Q^{FB}$ and welfare $W^{FB}$ is presented in Table 1 and derived in Appendix 1.

**Table 1:** Results for the FB case

	FB allocation
Subsidy	$p\left( 1-\rho _{j}^{FB}\right) =\theta \left( z_{j}+kz_{-j}\right)$, $j=1,2$
Quantities	$Q_j^{FB} = \frac{1}{2} \left( 1-\frac{p}{\beta \theta } +\frac{z_j+kz_{-j}}{\beta } \right)$, $j=1,2$
	$$Q^{FB}=1-\frac{p}{\theta \beta }+\frac{(1+k)\left( z_{1}+z_{2}\right) }{2\beta }$$
Welfare	$W^{FB} =Y-p+\theta \left( \frac{\beta }{2}+\frac{\left( z_{1}+z_{2}\right) \left( 1+k\right) }{2}\right) +\frac{p^{2}- \left( z_{1}+z_{2}\right) \left( 1+k\right) p\theta }{2\beta \theta } +\theta \frac{\left( z_{1}+kz_{2}\right) ^{2}+\left( z_{2}+kz_{1}\right) ^{2}}{4\beta }$

The first line shows the price subsidy the planner should provide. As expected, it depends on the utility generated by the public good characteristics, gross of the spillover it creates.

The optimal total quantity reflects the three components that characterise the impure public good: $1-\frac{p}{\theta \beta }$ is the demand for its private good characteristic, while $\frac{(1+k)(z_{1}+z_{2})}{2\beta }$ is the willingness to pay for its public good aspect, gross of the spillovers effects.

$W^{FB}$ represents the ideal level of welfare, given preferences and resources, but this outcome cannot be obtained. CG provision implies that only the good with the lowest quality (in terms of utility), i.e. $\theta =1$ is supplied and that at central level $z_1$ and $z_2$ cannot be observed. On the other hand, LGs produce the goods with highest utility, but they do not take spillovers into consideration. CG may influence the decisions of lower tiers, but does not have enough information to define an optimal policy. In this environment it is necessary to find the second best solution that allows the minimum welfare loss with respect to the FB solution. In what follows we will analyse and compare two alternatives:

- *Centralisation.* CG produces the less productive variety of *y*. The quantity is uniform across regions and it is set according to estimated preferences for the good;
- *Devolution.* CG delegates the production of *y* to lower government tiers (LG). A matching grant (King 1984) is supplied by CG to reduce the negative effects of spillovers.

## Centralisation

Let’s examine the case where CG produces the goods with lower productivity ($\theta _C=1$). Since $z_{j}$ cannot be observed, we assume that an expected estimate *z* is used to determine the optimal provision. CG has to set the optimal subsidy that maximises welfare in this context. The optimal subsidy and quantities can be found by substituting $\theta$ with $\theta _C = 1$ and $z_1=z_2= z$ in the formulas in the first two rows of Table 1 and are presented in Table 2. The welfare $W^C$ is obtained by substituting the optimal user charges $p\rho _1^C$ and $p\rho _2^C$ in (3).

**Table 2:** Results for the centralised solution

	Centralised allocation
Subsidy	$p\left( 1-\rho _{j}^{C}\right) =z(1+k)$, $j=1,2$
Quantities	$Q_j^{C} = \frac{1}{2} \left( 1-\frac{p}{\beta } +\frac{(1+k)z}{\beta } \right)$, $j=1,2$
	$$Q^{C}=1-\frac{p}{\beta }+\frac{(1+k)z}{\beta }$$
Welfare	$W^{C}=Y-p+\frac{\beta +\left( z_{1}+z_{2}\right) (1+k)}{2} +\left( z-\frac{z_{1}+z_{2}}{2}\right) \frac{p(1+k)-z(1+k)^{2}}{\beta } +\frac{1}{2}\frac{(p-z(1+k))^{2}}{\beta }$

Let’s compare the results in Tables 1 and 2. Even when asymmetry of information is minimal, e.g. when $z_1=z_2=z$, the subsidy is lower than in FB: for the quality provided at central level the willingness to pay is lower and thus the subsidy. This in turn means that the quantity of good produced is not optimal. As a consequence, welfare is not maximised and $W^{FB}-W^{C}$ measures the welfare loss.

## Devolution

In the previous section we showed that centralised provision does not allow to reach the FB allocation; here we consider the alternative solution of devolving production to lower tiers that know local preferences and can provide a good that fits best user needs. In this section we consider and compare two possible alternatives:

- “pure devolution” where LGs are solely responsible for the provision of the impure public good (the case will be indicated by the letter F);
- devolution where LGs provide the good, but CG influences their decisions using a matching grant.^9^

### Pure devolution

If the goods produced at local level were local public goods, devolution would always allow to reach the welfare level of FB (Oates 1972). However, the presence of spillovers means that also devolution is a second best option. In this case each Local Government (LG) maximises its utility function, but it does not take into account the accrued utility experienced by users in other local authorities. As for centralisation, each LG has to find the optimal user charge $p_u^j=p\nu _{j}$ that maximises the aggregate utility of jurisdiction *j*. The subsidy will be financed using a (local) linear income tax at rate $t_j$; the budget constraint in this case is

$$\frac{Y}{2} t_j = \frac{1}{2} \left( 1-\nu _j\right) p \left( 1-\frac{\nu _j p}{\beta \theta } \right) .$$

LG has to find the value of $\nu _j$ that maximises the following objective function:

$$\frac{1}{2} \int _{\underline{M}}^{\overline{M}}\left( \int _{0}^{\beta } U^{j}\left( M;\alpha ;p_u^j;z_j\right) \frac{1}{\beta } \text {d}\alpha \right) f(M)\,\text {d}M.$$

Table 3 shows the results derived in Appendix 2 in terms of the optimal subsidy $p(1-\nu _{j}^{F})$, quantities $Q_j^{F}$, total quantity $Q^F$ and total welfare $W^{F}$.

**Table 3:** Results for devolution with no matching grant

	“Pure” devolution
Subsidy	$p\left( 1-\nu _{j}^F\right) =\theta z_{j}$, $j=1,2$
Quantities	$Q_j^{F}=\frac{1}{2} \left( 1-\frac{p}{\theta \beta } +\frac{ z_j}{\beta }\right)$, $j=1,2$
	$$Q^{F}=1-\frac{p}{\theta \beta }+\frac{z_{1}+z_{2}}{2\beta }$$
Welfare	$W^{F}=Y-p+\theta \left( \frac{\beta }{2}+\frac{(z_{1}+z_{2})(1+k)}{2}\right) +\frac{p^{2}-(z_{1}+z_{2})(1+k)p\theta }{2\beta \theta } +\theta \, \frac{z_{1}^{2}+4kz_{1}z_{2}+z_{2}^{2}}{4\beta }$

Comparing Table 1 with Table 3 it is straightforward to see that the two results coincide for $k=0$, i.e. when there are no spillovers. In all the other cases, the subsidy is too low, total quantity falls short of the optimal level, and the welfare level attained is lower than in FB.

This is the first result of our model: the findings of the traditional literature on fiscal federalism are valid also for the provision of impure public goods, provided that there are no spillovers among regions. In all the other cases, “pure” devolution produces a welfare loss which is equal to $\Delta W_{F}=W^{FB}-W^F=\frac{k^{2}\theta }{4\beta }(z_{1}^{2}+z_{2}^{2})$.

### Devolution with a matching grant

CG may try to influence the choice of the local subsidy using a matching grant.^10^ This solution has some drawbacks: the asymmetry of information that prevents Central Government from providing the optimal quantity of *y* may also influence the optimal matching grant setting decision, for two main reasons:

- coordination problems, fiscal illusion and spillovers: due to the specific characteristics of *y*, any change in $Q_{j}$ affects the level of utility in authority $-$*j* and its decision on $Q_{-j}$. The matching grant introduces another interdependence in decisions, because the level of local expenditure has an impact on the national tax rate, hence on the welfare of each jurisdiction. If local decision makers misperceive the effects of their actions, CG may be unable to attain FB, even when it can observe the reaction function of LGs;
- asymmetry of information: CG cannot observe local preferences parameters and the reaction function of each local government.

The environment is characterised by the following assumptions: *y* is subsidised by LG, which does not take into account the spillovers created by its production. CG influences the behaviour of LGs using a matching grant.

The timing of the game is as follows: (a) CG sets the grant to maximise total welfare using its beliefs on LGs’ behaviour and users’ preferences; (b) LGs set their reaction function and their local tax rate.

Although CG cannot observe some relevant parameters, LGs are followers, i.e. we rule out the possibility that they may act strategically in setting their reaction function.^11^ The problem can be solved through backward induction: in the first stage LG decisions and reactions to a grant setting are considered; in the second stage CG finds the optimal grant, given its information set.

The analytical model is presented in Appendix 3. In what follows we discuss the intuition behind the findings.

#### LG reaction function

LG receives a matching grant at rate $(1-\rho _{j})p$ from CG. If it thinks that the good should be further subsidised, it will introduce a supplementary subsidy at rate $\eta _{j}$, to be financed by a proportional tax on local income at rate $\tau _{j}$. The user charge for the service will be equal to $p(\rho _{j}-\eta _{j})$. LG decision has a twofold impact on total welfare: on the revenue side it will change the national tax rate; on the expenditure side it will alter the quantity of the impure public good. This is one of the novel elements of our model: given the nature of *y*, each LG has to foresee the behaviour of the other local authority and should take into account the impact of increasing its expenditure. These effects may not be correctly perceived by LGs. In our model we consider three alternative behaviours for the LGs.

- (FC) Each LG thinks that the other local authority will replicate the same strategy (full coordination—FC), i.e. it will subsidise local production by the same amount. On the revenue side the overall change in the national tax rate is taken into account and on the expenditure side for each *j* the quantity $Q_{-j}$ is updated accordingly.
- (PC) Each LG thinks that the other local government does not further subsidise the good (partial coordination—PC). In this case the quantity $Q_{-j}$ will not change, while a one-sided correction of the national tax rate is considered.
- (FR) Each LG thinks that the effects of its expenditure on the tax rate are marginal, so that its decision influences neither the rate, nor *Q* (free rider, FR). The latter hypothesis may not be reasonable in a model with two local authorities, but in a more general context where the number of jurisdictions is fairly large this behaviour may be quite plausible. It is interesting to note that this is the hypothesis that has been used by the traditional literature in defining grant in the presence of spillovers.

The detailed derivation of the formulas for the local subsidy $p\,\eta _j$ is presented in Appendix 3 and reported in Table 4. For each LG behaviour two values are reported, because a second source of asymmetry of information has to be taken into account. The actual subsidy set at local level also depends on the local preferences $z_{j}$, which cannot be observed; as in section “Centralisation” we assume that an estimate *z*, equal for both local authorities is used by CG to guess the subsidy level that will be set by LGs.

**Table 4:** Local subsidy in case of devolution with a matching grant

LG behaviour	Actual local subsidy	Subsidy estimated by CG
FC	$\theta z_j (1+k)-p(1-\rho _{j})$	$\theta z (1+k)-p(1-\rho _{j})$
PC	$z_j \theta -p\frac{1-\rho _{j}}{2}$	$z \theta -p\frac{1-\rho _{j}}{2}$
FR	$z_j \theta$	$z \theta$

From Table 4 we note that the subsidy set by a LG behaving as a FR is higher than that in the PC case, as one might expect. A “free rider” behaviour implies that the LG does not take into account the increase in the national tax rate that is action is causing, i.e. LG underestimate the tax price for good *y* and will be prepared to subsidise it at a higher rate. A more general conclusion cannot be drawn: the relative effects of spillovers and national matching grant will determine the result. The use of a matching grant may allow CG to improve on pure devolution only if the grant is set correctly, but CG can observe only a subset of the parameters. For this reason, it will have to devise a strategy to minimise the negative effects due to the lack of information.

#### Grant setting

In the second stage CG has to set the matching grant, based on his beliefs about the behaviour of the LGs and the estimation of the unobservable preferences. We assume that CG expects LGs to have the same behaviour (i.e. both are either FC, PC or FR), thus the matching grant will be equal for the two regions (i.e. $\rho =\rho _1=\rho _2$). The estimated reactions of the LGs (the subsidy in the last column in Table 4) are then used to determine the welfare function $\tilde{W}_{\text {B}}(\rho )$ for B=FC,PC,FR using (3). CG has to decide which of the LG reaction is more plausible and assigns a probability $\pi ^{\text {B}}$ to each of the three possible reactions; the matching grant $\rho$ will be then found by maximising the expected welfare $E(W) = \sum \nolimits _{\text {B}} \pi _{\text {B}} \tilde{W}_{\text {B}}(\rho )$. The analytic derivation of the optimal grant in the general case is presented in Appendix 3. The solution depends on the probabilities $\pi ^{\text {B}}$; in Table 5 the optimal subsidies for the following relevant cases are shown: CG believes that LGs will behave as either FC, PC or FR (i.e. $\pi ^{\text {B}}=1;\pi ^{-\text {B}}=0$ for each possible value of B) and the case where CG assigns equal probability to each of the behaviours, i.e. $\pi ^{\text {B}}=\frac{1}{3}$ for all B (the abbreviation “Equi” is used for this case). Note that if LGs act as FC, CG cannot influence the expected welfare and no matching grant will be used. For PC the grant is twice the size than for FR, as one might expect. Finally, if all the reactions functions are taken in consideration with equal weight (Equi) the optimal grant is slightly higher than for FR, but quite close. The traditional literature and most actual grant formulae use FR assumption to model the behaviour of LGs. FR behaviour has a boosting effect on expenditure; by assuming the worst scenario in terms of effects on expenditure, CG tries to reduce the negative impacts on its expenditure of LG choices.

**Table 5:** Matching grant

CG belief	Grant
FC	0
PC	2*kz* $\theta$
FR	$kz\theta$
Equi	$\frac{6}{5} k z \theta$

Ex-post welfare analysis

Once the grant has been set by CG, the LGs will further subsidise the good following the scheme presented in the second column in Table 4. In general, for each case of the grant setting, three different reactions of the LGs are possible. The state contingent solution is presented in Table 8 in Appendix 3. In what follows we examine the results from a more qualitative point of view; the discussion will be supported by the graphical visualisation of the main findings.

Let us start by examining the case where CG assumes that LGs reaction is of the “fully coordinated” type (FC). It turns out that the difference of the user charge with respect to the FB case does not depend on CG grant, which will therefore be set to zero. If the action of the LGs is either PC or FR, the outcome of the “pure” devolution case is replicated. The difference in quantities with respect to the FB case is:

$$\Delta Q^F_{j} = \frac{k \,z_{-j}}{2 \beta } , \quad \Delta Q^F = \frac{k}{\beta } \frac{z_1+z_2}{2}$$

and the welfare difference is:

$$\Delta W_F = \frac{k^2 \theta }{4\beta } \left( z_1^2+z_2^2\right).$$

Note also that, if the reaction of LGs is FC, the outcome does not depend upon the action of CG: the total quantity is equal to the one in FB, but if the preferences in the two regions differ, it is not correctly distributed among them. In fact the difference in each region is $\frac{k}{2\beta } (z_j-z_{-j})$ and this causes a welfare loss with respect to the FB case equal to $\frac{k^2 \theta }{2\beta } (z_1-z_2)^2$.

If instead LGs reaction is either PC or FR, CG can reduce the difference in the subsidy and total quantity using a matching grant, by an amount that depends on *z*. If CG beliefs are fulfilled, the result is the same in both cases (PC and FR) and, if $z=\frac{z_{1}+z_{2}}{2}$ the total quantity produced is optimal, while the welfare loss is half the one obtained when LGs react as FC. Again, the total quantity is optimal, but its distribution across local authorities is different from FB and the welfare level is lower.

**Fig. 1:**
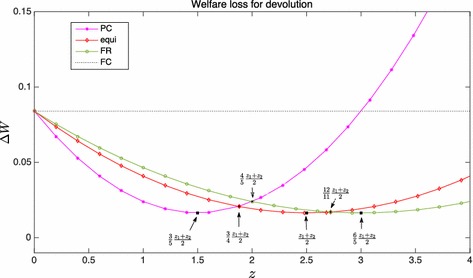
Comparison of the mean welfare loss produced by the matching grant for various levels of *z* and under the different assumptions on the behaviour of LGs

When the reaction of the local authorities is uncertain, the analysis has to be carried out by comparing the mean value of the welfare functions. In all cases the term $\frac{k^{2}\theta }{\beta }$ can be factored out, and the comparison only depends on *z*, i.e. on the quality of the information that CG has on the preferences of the two regions. The analytical comparison can be made by standard algebraic calculations; Fig. 1 illustrates the results by showing the relative position of the average welfare losses (wrt FB) under the different assumptions of CG about the reaction function of LGs. With the exception of the FC case, welfare losses are convex in *z*. The assumption that LG reacts as PC minimises the welfare loss only if CG considerably underestimates the average $\bar{z}=\frac{z_{1}+z_{2}}{2}$ of local preferences $\left(z\,\hbox{<}\,\frac{3}{4}\bar{z}\right)$. Let us call $\Delta W_{\text {FR}}$ and $\Delta W_{\text {Equi}}$ the ex-post average welfare differences (the last column in Table 8) under the two assumptions “FR” and “Equi” of CG on the behaviour of LG. If *z* ranges in $\left[ \frac{3}{4}\bar{z},\frac{12}{11}\bar{z}\right]$ then $\Delta W_{\text {Equi}}$ is the lowest and has a minimum for $z=\bar{z}$. The welfare loss that can be expected using FR performs better for higher values of *z* and has its minimum value for $z=\frac{6}{5}\bar{z}$.

$\Delta W_{\text {Equi}}$ increases at a higher rate than $\Delta W_{\text {FR}}$ as the distance of *z* from $\bar{z}$ increases because the grant under FR is lower than with the other assumptions. At the left of $\bar{z}$ the grant may be too low to make local authorities react optimally and local preferences are underestimated. At the right of $\bar{z}$ the two effects may offset each other. The two welfare losses are equal for $z=\frac{12}{11}\bar{z}$; this implies that for values of *z* lower than or very close to the mean, using a grant that minimises the expected welfare loss is preferred to assuming that LGs are free riders. In setting the matching grant, most traditional literature on fiscal federalism implicitly or explicitly^12^ assumes that CG may observe local preference on average and sets the grant as if local authorities were free riders. If CG can observe the mean of true preferences (i.e. $z=\bar{z}$), it should use the grant that minimises the welfare loss, but the mistake made using FR is small.

The comparison with the “pure devolution” case is less clearcut: the welfare loss $\Delta W_F$ does not depend on *z* and in a graphical comparison similar to the one in Fig. 1 it is represented as a horizontal line. Its relative position in the picture depends on the ratio of the preferences in the two regions. If the ratio is not too high, $\Delta W_F > \Delta W_{FC}$ and obviously any solution with a matching grant is preferable. As the ratio gets larger, “pure devolution” could anyhow be a viable option only in extreme cases, where either *z* greatly underestimates $\bar{z}$, or if the magnitude of the two parameters $z_1$ and $z_2$ is extremely different.^13^ Thus, ruling out unrepresentative cases, we can then conclude that a matching grant generally improves welfare, i.e. in the presence of spillovers it is optimal for CG to induce LGs to change their expenditure patterns using a matching grant.

## Centralisation versus devolution

Centralisation correctly takes into account spillovers, but it never allows to reach FB because goods produced in centralisation have the lowest quality level $(\theta _c=1).$ On the other hand, devolution has drawbacks in terms of coordination, because LGs are unable to take into account the utility that users outside their jurisdiction attaches to their production. In the previous section we have shown that “pure devolution” is not optimal: CG intervention with a matching grant improves welfare. In this section we compare centralisation with this model of devolution. The welfare loss in the centralised decision depends on the quality gap ($\theta$) and on the difference between the true preferences for the public good ($z_{1}$ and $z_{2}$), and the estimate used by CG (*z*). The one in devolution depends on the information CG has on local preference and LG’s reaction to the grant.

**Table 6:** Welfare loss comparison

Regime	Expected welfare loss
Centralisation	$\Delta W_{\text {C}}=\left( \theta -1\right) \left( \frac{\beta +\left( z_{1}+z_{2}\right) \left( 1+k\right) }{2}-\frac{p^{2}}{2\beta \theta }\right) +\theta \frac{(z_{1}+kz_{2})^{2}+(z_{2}+kz_{1})^{2}}{4\beta } +\frac{z(z-z_{1}-z_{2})(1+k)^{2}}{2\beta }$
Devolution with matching grant	
Devolution (Equi)	$\Delta W_{\text {Equi}}=\frac{k^{2}\theta }{\beta }\left( \frac{1}{3}(z_{1}^{2}-z_{1}z_{2}+z_{2}^{2})+\frac{3}{10}z[z-(z_{1}+z_{2})]\right)$
Devolution (FR)	$\Delta W_{\text {FR}}=\frac{k^{2}\theta }{\beta }\left( \frac{1}{3}(z_{1}^{2}-z_{1}z_{2}+z_{2}^{2})+\frac{1}{4}z[\frac{5}{6}z-(z_{1}+z_{2})]\right)$
Devolution without matching grant	
Pure devolution	$\Delta W_F=\frac{k^{2}\theta }{4\beta }(z_{1}^2+z_{2}^2)$

Table 6 summarises the welfare losses under the various assumptions. The welfare loss for centralisation derives from under-provision and from the lower utility each unit of impure public good produces; for devolution the loss derives from the provision of the wrong quantity of impure public good. The welfare loss for devolution is zero if $k=0$: if the goods produces spillovers $(k > 0)$ there might be scope for centralised provision.

The two parameters $\theta$ and *k* are thus fundamental in the comparison between $\Delta W_{\text {C}}$, the welfare loss for centralisation, and $\Delta W_{\text {D}}$, the welfare loss for devolution (the latter depends on CG’s information set and we denote it generally by a subscript D). As for the dependence on $\theta$, the function $\Delta W_{\text {C}}$ is increasing and convex, while $\Delta W_{\text {D}}$ is linear in this variable; both are quadratic polynomials in *k*. For $\Delta W_{\text {C}}$, an increase in *k* reduces the loss deriving from choosing a uniform level of provision in the two local authorities, but as $\theta$ increases, the loss caused by producing a goods of relatively lower quality increases. The prevailing effect depends on the values of *k* and $\theta$.

If we assume that CG can observe average local preferences, i.e. $z=\frac{z_{1}+z_{2}}{2}$, the lowest value for $\Delta W_{\text {D}}$ is equal to $\Delta W_{\text {Equi}}$. In Appendix 4 it is shown that the welfare loss increases more rapidly for centralisation than for (the best case of) devolution. Therefore devolution is the best option for any $\theta$ whenever $\Delta W_{\text {C}}\ge \Delta W_{\text {Equi}}$ for $\theta =1$. In all other cases there exists a (unique but depending on *k*) value $\theta ^{*}>1$ for which if $\theta <\theta ^{*}$ centralisation performs better than (the best case of) devolution. The sign of $\Delta W_{\text {C}}\ge \Delta W_{\text {Equi}}$ depends on *k* and the following can be proved (see Appendix 4):

$$\begin{gathered} {\text{if }}\,k \le k^{*} \quad {\text{then}}\quad \Delta W_{{\text{C}}} > \Delta W_{{{\text{Equi}}}} \quad \forall \theta > 1; \hfill \\ {\text{if }}\,k > k^{*} \quad {\text{then}}\quad \left\{ {\begin{array}{*{20}l} {\Delta W_{{\text{C}}} < \Delta W_{{{\text{Equi}}}} \quad 1 \le \theta < \theta ^{*} ,} \hfill \\ {\Delta W_{{\text{C}}} = \Delta W_{{{\text{Equi}}}} \quad \theta = \theta ^{*} ,} \hfill \\ {\Delta W_{{\text{C}}} > \Delta W_{{{\text{Equi}}}} \quad \theta > \theta ^{*} ,} \hfill \\ \end{array} } \right. \hfill \\ \end{gathered}$$

The value of $k^{*}$ is shown in (16); the one for $\theta ^{*}$ can be found explicitly, but the expression is quite cumbersome.

The above analysis has the following economic interpretation: for $\theta =1$, the welfare loss in centralisation is due only to the use of *z* instead of $z_{1}$ and $z_{2}$, which becomes less and less important as *k* increases. At the same time the loss in devolution increases if spillovers become important. For sufficiently high values of *k* centralisation should be preferred. On the other hand, for a fixed value of *k*, an increase in $\theta$ has the same effect on both welfare losses, but has a comparatively greater effect on $\Delta W_{\text {C}}$ and this gradually (as the spillover increases) offsets the gain produced by a mitigation of the mistake produced by the uniform distribution of the goods between the two regions.

**Fig. 2:**
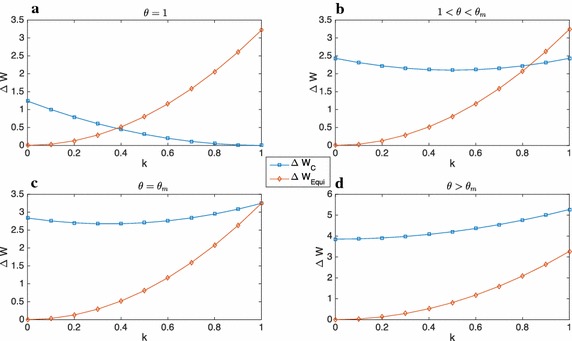
Dependence on *k* of the welfare losses in centralisation and devolution for $z=\bar{z}$ for different values of $\theta$. For $\theta =\theta _{m}$ the welfare losses in $k=1$ are equal. **a** $\theta =1$, **b** $1 < \theta < \theta _{m}$, **c** $\theta = \theta _{m}$, **d** $\theta > \theta _{m}$

**Fig. 3:**
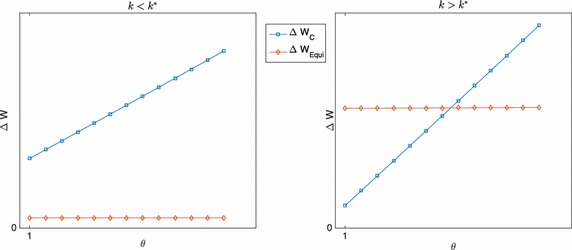
Dependence on $\theta$ of the welfare losses in centralisation and devolution for $z=\bar{z}$ for $k<k^{*}$ and $k>k^{*}$

The dependence of the difference of welfare levels on both *k* and $\theta$ is depicted in Figs. 2 and 3, while a numerical example is presented in Table 7. In Fig. 2 one can observe that as $\theta$ increases [i.e. passing from the graph (a–d)] the scope for centralisation gradually reduces. In (d) the two welfare losses are depicted for $\theta >\theta _{m}$, where $\theta _{m}$ is the value of $\theta ^{*}$ for $k=1$: in this case devolution always performs better than centralisation.

From the above results it also follows that the abscissa of the intercept between $\Delta W_{\text {C}}$ and $\Delta W_{\text {FR}}$ as functions of $\theta$ is smaller that $\theta ^{*}$. Thus, assuming that LGs are free riders leaves more scope for devolution than what would be optimal.

If CG is able to predict the behaviour, but cannot observe preferences, the minimum productivity differential $\theta ^{*}$ for which devolution should be preferred to centralisation may be evaluated using the same approach presented above. In this case, given that in devolution the reduction in welfare is lower than for the general case, $\theta ^{*}$ will be lower than in the model just presented, but there will still be an area in which devolution is not the best option.

These results are confirmed by the numerical simulations presented in Table 7. The first two simulations shows the effect of the spillover *k* on the optimal choice for a good whose public good component is relatively high. Preferences are rather homogeneous across regions and the quality produced in devolution is substantially higher than in centralisation. In this case devolution with a matching grant (Equi) is the second best choice, but the loss produced by FR is rather low.

On the contrary, if we consider a case where the quality differential is not important and preferences are heterogeneous, the public good aspect is less significant and centralisation is the best choice. In both cases, *k* increases the distance between welfare losses.

## Conclusions

This paper studies the conditions under which devolution is a second best choice for the provision of goods and services are impure public goods with spillovers. Devolution calls for coordination in the actions of lower tiers to prevent a failure of the process, even when CG observes the relevant parameters and could use a matching grant to internalise spillovers.

In the more general case where CG cannot observe LG’s reaction function and local preferences, devolution may cause large welfare losses. Its size depends on the spillovers effect *k*, on the level of the productivity $\theta$ of the goods produced at local level and on how well CG can predict the parameters it cannot observe. Our main conclusion is that devolution should be used only if the productivity (in terms of utility) of the goods produced at local level is sufficiently high to counterbalance the welfare loss produced by coordination, asymmetry of information and spillover effects. In all the other cases a centralised solution might be more effective. The traditional results of the literature on fiscal federalism are replicated by our model: for a local public good $(k=0),$ the FB solution is centralisation. In this respect our model can be considered a generalisation of the framework proposed by the traditional literature. The second interesting result of our model is that CG correction through a matching grant is always welfare improving, provided the grant that minimises the expected loss is chosen.

From a policy point of view the results of our model show that in the presence of asymmetry of information CG has to balance autonomy with control and it may prefer the former to the latter also for welfare maximisation reasons. When the quality of the goods does not depend on the tier that produces it ($\theta$ close to 1), but spillovers are important ($k>k^{*}$), a centralised solution might be optimal in a second best environment. This is the choice that has been made by some countries like the UK where the provision of services like health and education are still very centralised and in general it may explain why the level of decentralisation in decision making is lower in Europe than in the US.

The work presented in this paper could be extended in several directions: first of all, redistribution policies could be considered by introducing a specific income distribution at national and local level. This point is very important because the interaction between equalisation grants and fiscal federalism may produce perverse effects and it may be one of the causes for failures in the fiscal federalism structure such as soft budget constraint policies (Breuillé and Vigneault 2010; Levaggi and Menoncin 2013). Secondly, political considerations could be introduced by assuming that political parties compete for votes on different objectives and in a different setting at national and local level.

## Appendix 1: Outcome of the FB ideal case

The FB solution is obtained solving the problem:

5 $$\begin{aligned}&\max _{\rho _{j}} \left[ Y - \frac{1}{2} \sum _{j=1}^{2}\left( 1-\rho _{j}\right) p \left( 1-\frac{\rho _j\,p}{\theta \beta }\right) +\sum _{j=1}^{2}\int _{\frac{\rho _{j}p}{\theta }}^{\beta } \left( \alpha \theta -\rho _{j}p\right) \frac{1}{2\beta }\text {d}\alpha \right. \nonumber \\&\left. \qquad +\,\frac{1}{2}\sum _{j=1}^{2}z_{j}\theta \left( 1-\frac{\rho _j\,p}{\theta \beta } + k \left( 1-\frac{\rho _{-j}\,p}{\theta \beta } \right) \right) \right] \end{aligned}$$

whose FOC is

$$\frac{1}{2}p\frac{-p+z_{j}\theta +\rho _{j}p+\theta k\,z_{-j}}{\beta \theta }=0, \quad j=1,2.$$

The optimal price subsidy is $p(1-\rho _{j}^{FB})= \theta (z_j+k z_{-j})$; the optimal quantity $Q_j^{FB}$ and optimal welfare $W^{FB}$ are then derived by substituting $\rho _{1}^{FB}$ and $\rho _{1}^{FB}$ in (1) and in the objective function in (5).

## Appendix 2: Solution of the pure devolution case

The maximisation problem in this case is:

6 $$\begin{aligned}&\max _{\nu _{j}} \left[ \frac{Y}{2} - \frac{1}{2} \left( 1-\nu _{j}\right) p \left( 1-\frac{\nu _{j} p}{\theta \beta }\right) +\int _{\frac{\nu _{j}p}{\theta }}^{\beta } \left( \alpha \theta -\nu _{j}p\right) \frac{1}{2\beta }\text {d}\alpha \nonumber \right. \\&\left. \quad \qquad +\,\frac{1}{2} z_{j}\theta \left( 1-\frac{\nu _j\,p}{\theta \beta } + 2 k Q_{-j} \right) \right] \end{aligned}$$

where $Q_{-j}$ is constant with respect to $\nu _j$. The FOC is

$$\frac{1}{2}p\frac{-p+z_{j}\theta +\nu _{j}p}{\beta \theta }=0,$$

thus the optimal subsidy is $p(1-\nu _{j}^{F})=\theta z_j$ and the quantity $Q_j^{F}=\frac{1}{2} \left( 1-\frac{p-\theta z_j}{\theta \beta }\right)$. The welfare $W^{F}$ is found by substituting the optimal values above in each objective function in (6) and summing the results.

## Appendix 3: Analysis of devolution with matching grant

### LG reaction function

The contribution of the matching grant at rate $(1-\rho _j)p$ from CG and the supplementary local subsidy at rate $\eta _j$ lower the user charge to the level $p_u^j=p( \rho _j -\eta _j )$. CG finances its subsidy using a proportional tax at rate $\bar{t}$, which is derived from the budget constraint (4). LG has to finance its subsidy using a proportional tax on local income at rate $\tau _{j}$ and the local budget constraint is:

7 $$\tau _{j}\frac{Y}{2} = \frac{1}{2} \eta _{j} p \left( 1 - \frac{p\left( \rho _{j}-\eta _{j}\right) }{\theta }\right) .$$

As discussed in “LG reaction function” section, LG decision has a twofold impact on total welfare: it changes the national tax rate and alters the quantity of available impure public good. CG has to form a belief on the behaviour of the LGs to estimate their reaction function and we focus our attention on three alternative options:

- (FC) every LG thinks that the other local authority sets the same subsidy $p \eta _j$, presumes that the national tax rate grows to:
  8 $$t^{\text {FC}}=\overline{t}+\left( 1-\rho _{j}\right) p^{2}\frac{\eta _{j}}{\beta \theta Y}$$
  and the quantity $Q_{-j}$ is $\frac{1}{2}\left( 1-\frac{p( \rho _{j}-\eta _{j})}{\theta \beta }\right)$.
- (PC) each LG thinks that the other local government does not further subsidise the good, thus assumes that the quantity $Q_{-j}$ does not depend on $\eta _j$ and the national tax rate is:
  9 $$t^{\text {PC}}=\overline{t}+\frac{1}{2}\left( 1-\rho _{j}\right) p^{2}\frac{\eta _{j}}{\beta \theta Y}.$$
- (FR) each LG thinks that the effects of its expenditure on the tax rate are marginal and the decision does not influence either the national tax rate, nor $Q{-j}$, thus
  10 $$t^{\text {FR}}=\overline{t}.$$

In order to reduce useless replications, we introduce some notation to write the optimisation problem for a generic LG behaviour in a compact form. For a superscript B, ranging in the set of the three different LG behaviours FC, PC and FR, we set $s^{\text {B}}=0$ for B=PC or FR and $s^{\text {FC}}=1$. Also, we denote by $\zeta _j$ the preference parameter for the public good utility of *y* (so that we can choose $\zeta _j=z_j$ when examining the exact reaction function of the LG and $\zeta _j=z$ when considering the reaction function estimated from CG).

11 $$\begin{aligned}&\max _{\eta _{j}} \left[ \frac{Y}{2}\left( 1-t^{\text {B}}-\tau _{j}\right) +\int _{\frac{p(\rho _{j}-\eta _{j})}{\theta }}^{\beta } \left( \alpha \theta -\left( \rho _{j}-\eta _{j}\right) p\right) \frac{1}{2\beta }\text {d}\alpha \nonumber \right. \\&\left. \quad \qquad +\,\zeta _j \theta \left( k\int _{\frac{p\,\rho _{j}}{\theta }}^{\beta }\frac{1}{2\beta }\text {d}\alpha +s^{\text {B}}k\int _{\frac{p(\rho _{j}-\eta _{j})}{\theta }}^{\frac{p\rho _{j}}{\theta }} \frac{1}{2\beta }\text {d}\alpha +\int _{\frac{p(\rho _{j}-\eta _{j})}{\theta }}^{\beta }\frac{1}{2\beta }\text {d}\alpha \right) \right] \nonumber \\&\quad t^{\text {B}} :(8); (9); (10)\quad \text {B}=\text {FC};\,\text {PC};\,\text {FR},\qquad \tau _{j}:\; \text {from }(7). \end{aligned}$$

The problem can be rewritten as:

$$\begin{aligned}&\max _{\eta _{j }} \left[ \frac{Y}{2}\left( 1-\bar{t}\right) - \frac{m}{2} \left( 1-\rho _{j}\right) p^{2}\frac{\eta _{j}}{\beta \theta } -\frac{1}{2} \eta _{j}\,p \left( 1 - \frac{p(\rho _{j}-\eta _{j})}{\theta \beta }\right) \right. \\&\quad \qquad +\int _{\frac{p\left( \rho _{j}-\eta _j\right) }{\theta }}^{\beta }\left( \alpha \theta -\left( \rho _{j}-\eta _{j}\right) p\right) \frac{1}{2\beta }\text {d}\alpha \\&\left. \quad \qquad +\,\zeta _j \theta \left( k\int _{\frac{p\,\rho _{j}}{\theta }}^{\beta }\frac{1}{2\beta }\text {d}\alpha +sk\int _{\frac{p(\rho _{j}-\eta _{j})}{\theta }}^{\frac{p\,\rho _{j}}{\theta }}\frac{1}{2\beta }\text {d}\alpha +\int _{\frac{p(\rho _{i}-\eta _{i})}{\theta }}^{\beta }\frac{1}{2\beta }\text {d}\alpha \right) \right] \end{aligned}$$

with: $m=2,\;s=1$ in FC, $m=1,\;s=0$ in PC and $m=0,\;s=0$ in FR. The FOC is:

$$\frac{1}{2}\zeta _j p\frac{ks+1}{\beta }-\frac{1}{2\beta }p^{2}\frac{m-m\rho _{i}+\eta _{j}}{\theta }=0,$$

so that the optimal value in non singular cases is given by

$$\eta _{i}^{\text {B}}=\eta _{i}^{\text {B}}(\rho _j;\zeta _j) =\frac{\zeta _j \theta (1+ks)}{p}-\frac{m}{2}(1-\rho _{j}).$$

Substituting the values for *m* and *s* for the three cases we obtain:

12a $$\eta _{j}^{\text {FC}} (\rho _j;\zeta _j)=\frac{\theta \zeta _j (1+k)}{p}-(1-\rho _{j});$$

12b $$\eta _{j}^{\text {PC}} (\rho _j;\zeta _j)=\frac{\zeta _j \theta }{p}-\frac{1-\rho _{j}}{2};$$

12c $$\eta _{j}^{\text {FR}} (\rho _j;\zeta _j)=\frac{\zeta _j \theta }{p}.$$

### Central Government grant

We assume that CG expects LGs to have the same behaviour and assigns a probability $\pi ^{\text {B}}$ to each of the three possible reactions (FC, PC and FR). CG will choose a grant $\rho$ equal for the two regions so that the expected welfare is maximised. The problem is thus written as:

13 $$\begin{aligned}&\max _{\rho } \sum _{\text {B=FC,PC,FR}} \pi ^{\text {B}} \left( Y\left( 1-t^{\text {B}}-\tau ^{\text {B}}\right) +\int _{\frac{\left( \rho -\eta ^{\text {B}}\right) p}{\theta }}^{\beta } \left( \alpha \theta -\left( \rho -\eta ^{\text {B}}\right) p\right) \frac{1}{\beta } \text {d}\alpha \right. \\&\left. \quad \qquad +\,z(1+k)\theta \int _{\frac{\left( \rho -\eta ^{\text {B}}\right) p}{\theta }}^{\beta } \frac{1}{\beta } \text {d}\alpha \right) \nonumber \\&t^{\text {B}} =\frac{p}{\beta \theta }\frac{(1-\rho )\left( \beta \theta -p\left( \rho -\eta ^{\text {B}}\right) \right) }{Y};\quad \tau ^{\text {B}} =\eta ^{\text {B}}p\frac{\beta \theta -p\left( \rho -\eta ^{\text {B}}\right) }{\beta \theta Y};\nonumber \\&\eta ^{\text {FC}} =\frac{\theta z(1+k)}{p}-(1-\rho );\quad \eta ^{\text {PC}}=\frac{z\theta }{p}-\frac{1-\rho }{2};\quad \eta ^{\text {FR}}=\frac{z\theta }{p}.\nonumber \end{aligned}$$

The derivative with respect to $\rho$ is:

$$\frac{p^{2}(1-\rho )}{4}\frac{4\pi ^{\text {FR}}+\pi ^{\text {PC}}}{\beta \theta } -\frac{zkp}{4}\frac{4\pi ^{\text {FR}}+2\pi ^{\text {PC}}}{\beta }.$$

If $\pi ^{\text {FR}}=\pi ^{\text {PC}}=0$ (and thus $\pi ^{\text {FC}}=1$) the welfare does not depend on $\rho$, i.e. CG cannot influence the welfare using a matching grant, thus $1-\rho$ will be set to zero. In all the other cases the optimal grant is equal to:

14 $$p\left( 1-\rho ^{*}\right) =kz\theta \left( 1+\frac{\pi ^{\text {PC}}}{\pi ^{\text {PC}}+4\pi ^{\text {FR}}}\right) .$$

### Outcome

The setting of the grant depends on the probabilities $\pi ^{\text {B}}$; in the analysis the four following cases will be taken into consideration: CG believes that LGs will behave as either FC, PC or FR (i.e. $\pi ^{\text {B}}=1;\pi ^{-\text {B}}=0$ for each possible value of B) and the case where CG assigns equal probability to each of the behaviours, i.e. $\pi ^{\text {B}}=\frac{1}{3}$ for all B (the abbreviation “Equi” is used for this case).

Once the grant has been set by CG, the LGs will further subsidise the good following the scheme presented in (12). In general terms, if we define $\gamma _{j}=p(1-\rho _{j}+\eta _{j})$, since

$$Q_j = \int _{\frac{p-\gamma _{i}}{\theta }}^{\beta }\frac{1}{2\beta }\text {d}\alpha = \frac{1}{2} \left( 1-\frac{p}{\beta \theta }+\frac{\gamma _j}{\beta \theta }\right) ,$$

we have

$$Q=Q_1+Q_2=1-\frac{p}{\beta \theta }+\frac{1}{\beta \theta }\frac{\gamma _{1}+\gamma _{2}}{2}$$

and

$$\begin{aligned} W&=Y-p+\frac{\beta \theta }{2}+\frac{p^{2}}{2\beta \theta }+\frac{1}{2\beta }(1+k)(z_{1}+z_{2})(\beta \theta -p) -\frac{1}{4\beta \theta }\left( \gamma _{1}^{2}+\gamma _{2}^{2}\right) \\&\quad +\,\frac{1}{2\beta }\left[ \gamma _{1}\left( z_{1}+kz_{2}\right) +\gamma _{2}\left( z_{1}+kz_{2}\right) \right] \end{aligned}$$

Since in this notation we have $\gamma _{j}^{\text {FB}}=\theta (z_{j}+kz_{-j})$, for any two couples $(\tilde{\gamma }_{1},\tilde{\gamma }_{2})$, $(\bar{\gamma }_{1},\bar{\gamma }_{2})$ the welfare difference can be written as

$$\tilde{W}-\bar{W}=\frac{1}{2\beta \theta }\sum _{j=1}^{2}\left( \bar{\gamma _{j}}-\tilde{\gamma _{j}}\right) \left( \frac{\bar{\gamma _{j}}+\tilde{\gamma _{j}}}{2}-\gamma _{j}^{\text {FB}}\right)$$

and the total quantity and welfare differences wrt the FB case amount to:

$$\begin{aligned} Q^{\text {FB}}-Q&=\frac{1}{\beta \theta }\left( \frac{\gamma _{1}^{\text {FB}} +\gamma _{2}^{\text {FB}}}{2}-\frac{\gamma _{1}+\gamma _{2}}{2}\right) \\ W^{\text {FB}}-W&=\frac{1}{4\beta \theta }\sum _{j=1}^{2}\left( \gamma _{j}-\gamma _{j}^{FB}\right) ^{2}. \end{aligned}$$

For each case of the grant setting, three different reactions of the LGs are possible. Ex post, the average welfare loss wrt FB is:

15 $$\Delta W=\frac{1}{12\beta \theta }\sum _{\text {B},j}\left( \gamma _{j}^{\text {B}}-\gamma _{j}^{\text {FB}}\right) ^{2},$$

where $\gamma _{j}^{\text {B}}=p(1-\rho ^{*}+\eta _{j}^{B}(\rho ^{*}))$ is the unit subsidy.

The overall state contingent solution is presented in detail in Table 8. The first column reports CG beliefs about LG’s reaction function;^14^ then for each case in the second column are listed the three possible LG behaviours. Reading further from left to right, for each (ex-post) case (CG belief, LG behaviour) the differences with respect to the FB case of the total subsidy $\gamma _j$ for region *j*, the total produced quantity *Q* and the welfare are shown. Finally, the last column presents, for each possible entry of the first column, the mean ex-post welfare loss. Lines in bolditalics refer to the case where CG beliefs are fulfilled and the only mistake that CG makes is due to its imperfect observation of local preferences.

**Table 7:** Numerical simulation

	$p^1_u$	$p^2_u$	$Q_{1}$	$Q_{2}$	*Q*	*t*	$\tau _{1}$	$\tau _{2}$	$t+\tau _{1}$	$t+\tau _{2}$	*W*
$Y=100$; $p=80$; $\theta =1.1$; $\beta =80$; $z_{1}=25$; $z_{2}=35$; $k=0.5$; $z=30$											
FB	33.250	27.750	0.311	0.342	0.653	0.324	0.000	0.000	0.324	0.324	118.828
C	35.000	35.000	0.301	0.301	0.602	0.271	0.000	0.000	0.271	0.271	112.656
F	52.500	41.500	0.202	0.264	0.466	0.000	0.111	0.203	0.111	0.203	117.239
Equi	38.017	38.017	0.284	0.284	0.568	0.112	0.126	0.126	0.238	0.238	118.639
FR	39.667	39.667	0.275	0.275	0.549	0.091	0.131	0.131	0.222	0.222	118.614
$Y=100$; $p=80$; $\theta =1.1$; $\beta =80$; $z_{1}=25$; $z_{2}=35$; $k=0.75$; $z=30$											
FB	23.625	20.875	0.366	0.381	0.747	0.432	0.000	0.000	0.432	0.432	124.574
C	27.500	27.500	0.344	0.344	0.688	0.361	0.000	0.000	0.361	0.361	117.227
F	52.500	41.500	0.202	0.264	0.466	0.000	0.111	0.203	0.111	0.203	120.996
Equi	30.775	30.775	0.325	0.325	0.650	0.193	0.127	0.127	0.320	0.320	124.148
FR	33.250	33.250	0.311	0.311	0.622	0.154	0.137	0.137	0.291	0.291	124.090
$Y=100$; $p=80$; $\theta =1.01$; $\beta =80$; $z_{1}=10$; $z_{2}=45$; $k=0.5$; $z=27.5$											
FB	47.175	29.500	0.208	0.317	0.526	0.229	0.000	0.000	0.229	0.229	111.641
C	38.750	38.750	0.260	0.260	0.520	0.215	0.000	0.000	0.215	0.215	110.635
F	69.900	34.550	0.067	0.286	0.354	0.000	0.014	0.260	0.014	0.260	109.964
Equi	59.884	59.884	0.129	0.129	0.259	0.043	0.009	0.009	0.052	0.052	110.595
FR	61.273	61.273	0.121	0.121	0.242	0.034	0.012	0.012	0.045	0.045	110.575
$Y=100$; $p=80$; $\theta =1.01$; $\beta =80$; $z_{1}=10$; $z_{2}=45$; $k=0.75$; $z=27.5$											
FB	35.813	26.975	0.278	0.333	0.611	0.300	0.000	0.000	0.300	0.300	115.226
C	31.875	31.875	0.303	0.303	0.606	0.291	0.000	0.000	0.291	0.291	114.475
F	69.900	34.550	0.067	0.286	0.354	0.000	0.014	0.260	0.014	0.260	111.453
Equi	54.876	54.876	0.160	0.160	0.321	0.080	0.000	0.000	0.081	0.081	112.872
FR	56.959	56.959	0.148	0.148	0.295	0.061	0.007	0.007	0.068	0.068	112.827

**Table 8:** Coordination problem under information asymmetry

CG	LG	$\Delta$-subsidy	$\Delta$-quantity	$\Delta$-welfare	Ex-post mean welfare loss
FC	*FC*	${\varvec{\theta }} {\varvec{k}}({\varvec{z}}_{{\varvec{j}}}-{\varvec{z}}_{-{\varvec{j}}})$	$0$	$\frac{{\varvec{k}}^{{\varvec{2}}}{\varvec{\theta }}}{{\varvec{2}} {\varvec{\beta }}}({\varvec{z}}_{{\varvec{1}}}-{\varvec{z}}_{{\varvec{2}}})^{{\varvec{2}}}$	$\frac{k^{2}\theta }{3\beta }\left( z_{1}^{2}+z_{2}^{2}-z_{1}z_{2}\right)$
	PC	$\theta kz_{-j}$	$\frac{k}{\beta }\frac{z_{1}+z_{2}}{2}$	$\frac{k^{2}\theta }{4\beta }\left( z_{1}^{2}+z_{2}^{2}\right)$	
	FR	$\theta kz_{-j}$	$\frac{k}{\beta }\frac{z_{1}+z_{2}}{2}$	$\frac{k^{2}\theta }{4\beta }\left( z_{1}^{2}+z_{2}^{2}\right)$	
PC	FC	$\theta k(z_{j}-z_{-j})$	0	$\frac{k^{2}\theta }{2\beta }(z_{1}-z_{2})^{2}$	$$\frac{k^{2}\theta }{\beta }\left( \begin{array}{c} \frac{1}{3}\left( z_{1}^{2}+z_{2}^{2}-z_{1}z_{2}\right) \\ +\,z\left[ \frac{5}{6}z-\frac{1}{2}(z_{1}+z_{2})\right] \end{array}\right)$$
	*PC*	${\varvec{\theta }} {\varvec{k(z}}_{-{\varvec{j}}} -{\varvec{z}})$	$\frac{{\varvec{k}}}{{\varvec{\beta }}} \left( \frac{{\varvec{z}}_{{\varvec{1}}} +{\varvec{z}}_{{\varvec{2}}}}{{\varvec{2}}}-{\varvec{z}}\right)$	$\frac{{\varvec{k}}^{{\varvec{2}}}{\varvec{\theta }}}{{\varvec{2\beta }}} \left[ \frac{({\varvec{z}}_{{\varvec{1}}}-{\varvec{z}})^{{\varvec{2}}}}{{\varvec{2}}}+\frac{({\varvec{z}}_{{\varvec{2}}}-{\varvec{z}})^{{\varvec{2}}}}{{\varvec{2}}} \right]$	
	FR	$\theta k(z_{-j}-2z)$	$\frac{k}{\beta }\left( \frac{z_{1}+z_{2}}{2}-2z\right)$	$\frac{k^{2}\theta }{2\beta }\left[ \frac{(z_{1}-2z)^{2}}{2}+\frac{(z_{2}-2z)^{2}}{2}\right]$	
FR	FC	$\theta k(z_{j}-z_{-j})$	0	$\frac{k^{2}\theta }{2\beta }(z_{2}-z_{1})^{2}$	$$\frac{k^{2}\theta }{\beta }\left( \begin{array}{c} \frac{1}{3}\left( z_{1}^{2}+z_{2}^{2}-z_{1}z_{2}\right) \\ +\,\frac{1}{4}z\left[ \frac{5}{6}z-(z_{1}+z_{2})\right] \end{array}\right)$$
	PC	$\theta k(z_{-j}-\frac{z}{2})$	$\frac{k}{\beta }\frac{z_{1}+z_{2}-z}{2}$	$\frac{k^{2}\theta }{4\beta }\left[ \frac{(2z_{1}-z)^{2}}{4}+\frac{(2z_{2}-z)^{2}}{4}\right]$	
	*FR*	${\varvec{\theta }} {\varvec{k}}({\varvec{z}}_{-{\varvec{j}}} -{\varvec{z}})$	$\frac{{\varvec{k}}}{{\varvec{\beta }}} \left( \frac{{\varvec{z}}_{{\varvec{1}}}+{\varvec{z}}_{{\varvec{2}}}}{{\varvec{2}}}-{\varvec{z}}\right)$	$\frac{{\varvec{k}}^{{\varvec{2}}}{\varvec{\theta }}}{{\varvec{2}}{\varvec{\beta }}} \left[ \frac{({\varvec{z}}_{{\varvec{1}}}-{\varvec{z}})^{{\varvec{2}}}}{{\varvec{2}}}+\frac{({\varvec{z}}_{{\varvec{2}}}-{\varvec{z}})^{{\varvec{2}}}}{{\varvec{2}}}\right]$	
Equi	FC	$\theta k(z_{j}-z_{-j})$	0	$\frac{k^{2}\theta }{2\beta }(z_{1}-z_{2})^{2}$	$$\frac{k^{2}\theta }{\beta }\left( \begin{array}{c} \frac{1}{3}\left( z_{1}^{2}+z_{2}^{2}-z_{1}z_{2}\right) \\ +\,\frac{3}{10}z\left[ z-(z_{1}+z_{2})\right] \end{array}\right)$$
	PC	$\theta k\left( z_{-j}-\frac{3}{5}z\right)$	$\frac{k}{\beta }\left( \frac{z_{1}+z_{2}}{2}-\frac{3}{5}z\right)$	$\frac{k^{2}\theta }{4\beta }\left[ \left( z_{1}-\frac{3}{5}z\right) ^{2}+\left( z_{2}-\frac{3}{5}z\right) ^{2}\right]$	
	FR	$\theta k\left( z_{-j}-\frac{6}{5}z\right)$	$\frac{k}{\beta }\left( \frac{z_{1}+z_{2}}{2}-\frac{6}{5}z\right)$	$\frac{k^{2}\theta }{4\beta }\left[ \left( z_{1}-\frac{6}{5}z\right) ^{2}+\left( z_{2}-\frac{6}{5}z\right) ^{2}\right]$	

## Appendix 4: Centralisation versus devolution

From the expression of the welfare $W_C$ (Table 2) we have

$$\begin{aligned} \Delta W_{\text {C}}&= {} W^{\text {FB}}-W^{\text {C}}\\&= {} \left( \theta -1\right) \left( \frac{\beta +\left( z_{1}+z_{2}\right) \left( 1+k\right) }{2}-\frac{p^{2}}{2\beta \theta }\right) \\&\quad +\,\theta \frac{\left( z_{1}+kz_{2}\right) ^{2}+\left( z_{2}+kz_{1}\right) ^{2}}{4\beta }+\frac{z\left( z-z_{1}-z_{2}\right) \left( 1+k\right) ^{2}}{2\beta } \end{aligned}$$

thus

$$\frac{\partial \Delta W_{\text {C}}}{\partial \theta }=\frac{\beta }{2}+\frac{z_{1}+z_{2}}{2}(1+k)+\frac{(z_{1}+kz_{2})^{2}+(z_{2}+kz_{1})^{2}}{4\beta }-\frac{p^{2}}{2\beta \theta ^{2}}.$$

and since $\beta \theta >p$ the function $\Delta W_{\text {C}}$ is strictly increasing and convex in $\theta$.

From Table 8 we have

$$\frac{\partial \Delta W_{\text {Equi}}}{\partial \theta }=\frac{k^{2}}{\beta }\left( \frac{1}{3}\left( z_{1}^{2}-z_{1}z_{2}+z_{2}^{2}\right) +\frac{3}{10}z\left( z-(z_{1}+z_{2})\right) \right)$$

and

$$\frac{\partial \Delta W_{\text {FR}}}{\partial \theta }=\frac{k^{2}}{\beta }\left( \frac{1}{3}\left( z_{1}^{2}-z_{1}z_{2}+z_{2}^{2}\right) +\frac{1}{4}z\left( \frac{5}{6}z-(z_{1}+z_{2})\right) \right) ,$$

so that $\frac{\partial \Delta W_{\text {Equi}}}{\partial \theta }<\frac{\partial \Delta W_{\text {FR}}}{\partial \theta }$ whenever $z<\frac{6}{11}(z_{1}+z_{2})=\frac{12}{11}\frac{z_{1}+z_{2}}{2}$.

If CG is able to observe the mean and it can set $z=\frac{z_{1}+z_{2}}{2}$, since $k\in [0,1]$, by standard algebraic manipulations we have

$$\begin{aligned} \frac{\partial \Delta W_{\text {Equi}}}{\partial \theta }&=\frac{k^{2}}{4\beta }\left( z_{1}^{2}+z_{2}^{2}\right) +\frac{k^{2}}{120\beta }\left( z_{1}^{2}+z_{2}^{2}\right) -\frac{29k^{2}}{60\beta }z_{1}z_{2}\\ \frac{\partial \Delta W_{\text {FR}}}{\partial \theta }&=\frac{\partial \Delta W_{\text {Equi}}}{\partial \theta }+\frac{k^{2}}{480\beta }(z_{1}+z_{2})^{2}\\&=\frac{k^{2}}{4\beta }\left( z_{1}^{2}+z_{2}^{2}\right) +\frac{k^{2}}{96\beta }\left( z_{1}^{2}+z_{2}^{2}\right) -\frac{23k^{2}}{48\beta }z_{1}z_{2}\\&<\frac{1}{4\beta }\left[ (z_{1}+kz_{2})^{2}+(z_{2}+kz_{1})^{2}\right] <\frac{\partial \Delta W_{\text {C}}}{\partial \theta }.\\ \end{aligned}$$

The situation remains the same if $z=\nu \frac{z_{1}+z_{2}}{2}$ for $\nu \le \frac{12}{11}$. For higher values of $\nu$, as obvious also from Fig. 1, the relative positions of the two devolution cases have to be interchanged. Apart from this, unless $\nu$ is very large, the considerations done above for $\nu =1$ are still valid in the general case.

From an analytical point of view, it is obvious that devolution is the best option for all values of $\theta$ whenever $\Delta W_{\text {C}}\ge \Delta W_{\text {Equi}}$ for $\theta =1$. In all other cases there exists a (unique but depending on *k*) value $\theta ^{*}>1$ for which if $\theta <\theta ^{*}$ centralisation performs better than (the best case of) devolution. The sign of $\Delta W_{\text {C}}-\Delta W_{\text {Equi}}$ for $\theta =1$ depends in turn on the value of *k*. Since for $k=0$ we have $\Delta W_{\text {C}}>0=\Delta W_{\text {Equi}}$, while for $k=1$ it holds $\Delta W_{\text {C}}=0<\Delta W_{\text {Equi}}$, there exists a (unique) value $k^{*}$ such that $\Delta W_{\text {C}}>\Delta W_{\text {Equi}}$ whenever $k\in [0,k^{*})$ and $\Delta W_{\text {C}}<\Delta W_{\text {Equi}}$ if $k\in (k^{*},1]$. Thus:

$$\begin{gathered} {\text{if }}\,k \le k^{*} \quad {\text{then}}\quad \Delta W_{{\text{C}}} > \Delta W_{{{\text{Equi}}}} \quad \forall \theta > 1; \hfill \\ {\text{if }}\,k > k^{*} \quad {\text{then}}\quad \left\{ {\begin{array}{*{20}l} {\Delta W_{{\text{C}}} < \Delta W_{{{\text{Equi}}}} \quad 1 \le \theta < \theta ^{*} ,} \hfill \\ {\Delta W_{{\text{C}}} = \Delta W_{{{\text{Equi}}}} \quad \theta = \theta ^{*} ,} \hfill \\ {\Delta W_{{\text{C}}} > \Delta W_{{{\text{Equi}}}} \quad \theta > \theta ^{*} ,} \hfill \\ \end{array} } \right. \hfill \\ \end{gathered}$$

The value of $k^{*}$ can be found analytically and has the following expression:

16 $$\frac{\sqrt{15}\cdot |z_{1}-z_{2}|}{4}\cdot \frac{\sqrt{31(z_{1}-z_{2})^{2}+4z_{1}z_{2}} -\sqrt{15}\cdot |z_{1}-z_{2}|}{4(z_{1}-z_{2})^{2}+z_{1}z_{2}}.$$

## Footnotes

1 An impure public good is a commodity (or a service) that has the double characteristic of being an appropriable good (as private goods) and a public good. Let us for example consider education. Education improves utility of students in terms of future earnings/better working conditions (this is the private good element), but it also improves the utility of the community in terms of skilled workers/professionals available to society (this is the public good element). Another important class of impure public goods are the so called option goods, where the utility for society is derived from the availability of the good in case of need. A good example may be local transport. It produces utility for services users, but it improves also the utility of non users (hence of the whole community) in terms of being an available option in case of need (see Musgrave and Musgrave 1989).

2 For a review of the debate see Oates (2005).

3 The Netherlands, for example have decentralised about 93 % of expenditure on environmental protection and virtually nothing as concerns health. On the contrary Denmark has almost fully decentralised health care, but only 60 % of expenditure on environmental protection.

4 See Oates (2005) for a review.

5 We share this assumption with Wildasin (2001). In a more general setting, a reasonable assumption would be to assume that the two local authorities have a different average income. For the purpose of this analysis such assumption is irrelevant. Petretto (2000) and Levaggi and Menoncin (2014) present a model with these characteristics as regards the distribution of income.

6 The use of more general utility functions is presented in Levaggi (2010).

7 For example, let us consider local transport: the same number of bus services produces a different level of utility according to its hourly distribution within the day.

8 To understand this point, consider for example the case $\alpha =0$: these individuals do not buy *y*, but as long as $z_j$ is non negative, they attribute a value to the production of that good.

9 In the fiscal federalism literature matching grants are transfers from higher to lower government tiers designed to complement sub-national contributions. They have a form similar to a price subsidy.

10 A matching grant is a form of subsidy to LGs proportional to its expenditure. In other words, the use of a matching grant means that a fraction of the total cost for the provision of *y* is borne by CG.

11
Wildasin (2001) and Köthenbürger (2008) show how local government could play strategically in a game where the goods to be produced is a local public good with spillovers.

12 As in Tresch (2002).

13 In fact $\Delta W_F\ge \Delta W_{FC}$ whenever $0.268\approx 2-\sqrt{3} \le \frac{z_1}{z_2} \le 2+\sqrt{3}\approx 3.732$, while $\Delta W_F$ is lower than the minimum welfare loss obtained with some matching grant only if $\frac{z_1}{z_2} > 29+2 \sqrt{210} \approx 57.983$ or $\frac{z_1}{z_2} < 29-2 \sqrt{210}\approx 0.017$.

## References

[CR1] Akai N, Mikami K (2006). Fiscal decentralization and centralization under majority rule: a normative analysis. Econ Syst.

[CR2] Alcidi C, Giovannini A, Infelise F, Ferrer JN (2014) Division of powers between the European Union, member states, candidate and some potential candidate countries, and local and regional authorities: fiscal decentralisation or federalism. European Union, Bruxelles. doi:10.2863/11797

[CR3] Barrios S, Strobl E (2009). The dynamics of regional inequalities. Reg Sci Urban Econ.

[CR4] Besley T, Coate S (2003). Centralized versus decentralized provision of local public goods: a political economy approach. J Public Econ.

[CR5] Breuillé M-L, Vigneault M (2010). Overlapping soft budget constraints. J Urban Econ.

[CR6] Calsamiglia X, Garcia-Milà T, McGuire TJ (2006) Why do differences in the degree of fiscal decentralization endure? CESifo Working Paper Series 1877, CESifo Group, Univ Center for Economic Studies, Munich

[CR7] Costa-Font J, Greer S (2013). Federalism and decentralisation in European health and social care.

[CR8] Dziobek CH, Gutierrez Mangas CA, Kufa P (2011) Measuring fiscal decentralization—exploring the IMFs databases. Tech. Rep. Working Paper No. 11/126, IMF, Washington

[CR9] King D (1984). Fiscal tiers: the economics of multi-level government.

[CR10] Köthenbürger M (2008). Revisiting the “decentralization theorem”—on the role of externalities. J Urban Econ.

[CR11] Levaggi R (2002). Decentralised budgeting procedures for public expenditure. Public Financ Rev.

[CR12] Levaggi R (2010). From local to global public goods: how should we write the utility function. Econ Model.

[CR13] Levaggi R, Menoncin F (2013). Soft budget constraints in health care: evidence from Italy. Eur J Health Econ.

[CR14] Levaggi R, Menoncin F (2014). Health care expenditure decisions in the presence of devolution and equalisation grants. Int J Health Care Financ Econ.

[CR15] Musgrave RA, Musgrave PB (1989) Public finance in theory and practice. Finance series, McGraw-Hill, Singapore

[CR16] Oates WE (1972). Fiscal federalism.

[CR17] Oates WE (2005). Toward a second-generation theory of fiscal federalism. Int Tax Public Financ.

[CR01] OECD (2016) General government spending (indicator). doi:10.1787/a31cbf4d-en. Accessed 1 Mar 2016

[CR18] Ogawa H, Wildasin DE (2009). Think locally, act locally: spillovers, spillbacks and efficient decentralized policy making. Am Econ Rev.

[CR19] Petretto A (2000). On the cost-benefit of the regionalisation of the national health service. Econ Gov.

[CR20] Sacchi A, Salotti S (2014). How regional inequality affects fiscal decentralisation: accounting for the autonomy of subcentral governments. Environ Plan C Gov Policy.

[CR21] Snoddon T, Wen J-F (2003). Grants structure in an intergovernmental fiscal game. Econ Gov.

[CR22] Sorens J (2014). Does fiscal federalism promote regional inequality? An empirical analysis of the OECD, 1980–2005. Reg Stud.

[CR23] Tanzi V (2009). The future of fiscal federalism and the need for global government: a reply to Roland Vaubel. Eur J Polit Econ.

[CR24] Thieben U (2003). Fiscal decentralisation and economic growth in high-income OECD countries. Fisc Stud.

[CR25] Tresch RD (2002). Public finance: a normative view.

[CR26] Turati G, Montolio D, Piacenza M (2011) Fiscal decentralisation, private school funding, and students achievements. a tale from two roman catholic countries. Tech. Rep. Working Papers 2011/44, Institut d’Economia de Barcelona (IEB), Barcelona

[CR27] Vaubel R (2009). The future of fiscal federalism and the need for global government: a response to Vito Tanzi. Eur J Polit Econ.

[CR28] Weisner E (2003) Federalism in Latin American: from entitlements to markets. Inter-American Development Bank, Johns Hopkins University Press, Washington

[CR29] Wildasin D (2001) Externalities and bailouts: hard and soft budget constraints in intergovernmental fiscal relations. Public economics, EconWPA. http://www.EconPapers.repec.org/RePEc:wpa:wuwppe:0112002

[CR30] Wildasin DE (2004). The institutions of federalism: towards an analytical framework. Natl Tax J LVII.

